# Positivity-Preserving Hybridizable Discontinuous Galerkin Scheme for Solving PNP Model

**DOI:** 10.3390/e27111175

**Published:** 2025-11-20

**Authors:** Diana Morales, Zhiliang Xu

**Affiliations:** Department of Applied and Computational Mathematics and Statistics, University of Notre Dame, Notre Dame, IN 46556, USA; dmorale3@nd.edu

**Keywords:** Poisson-Nernst-Planck equation, hybridizable disconinuous Galerkin, positivity preserving, energy stability

## Abstract

We introduce a hybridizable discontinuous Galerkin (HDG) scheme for solving the Poisson–Nernst–Planck (PNP) equations. The log-density formulation as introduced by Metti et al. in their paper “Energetically stable discretizations for charge transport and electrokinetic models. J. Comput. Phys. 2016, 306, 1-18” is utilized to ensure the positivity of the densities of the charged particles. We further prove that our fully discrete scheme is energy stable and mass conserving. Numerical simulations are provided to demonstrate the accuracy of the scheme in one and two spatial dimensions. A derivation of an HDG-DG space–time scheme is given, with implementation and convergence analysis left to future work.

## 1. Introduction

The Poisson–Nernst–Planck (PNP) equations are one of the most investigated systems for ion transportation modeling [1] due to their ability to capture the intricacies of ion channels. A PNP system typically comprises two types of equations: a Poisson equation and Nernst–Planck equations. The Poisson equation models how the charge density creates electric potential and the Nernst–Planck equations model the diffusion of ions based on conservation laws [2]. Beyond ion channel modeling [1,3,4,5,6,7], PNP systems have been utilized for the modeling of semiconductors [8,9,10,11], electromembrane extraction [12,13], and batteries [14,15].

The study of PNP models provides quantitative and qualitative reasoning to help explain ion transport phenomena. Many numerical solvers have been introduced for solving the PNP system of equations [2,3,5,6,7,16,17,18]. Among them, discontinuous Galerkin (DG) methods have been a popular choice for solving the PNP equations. This is because DG methods have many established advantages over finite volume and finite difference schemes, such as flexibility in handling complex geometries, high-order accuracy, and hp-adaptivity.

In this work, we choose to utilize a hybridizable discontinuous Galerkin (HDG) method for our spatial discretization and implicit time discretization. The HDG method was pioneered by Cockburn et al. [19,20] and is known to retain the advantages of DG methods while reducing the computational cost. The computational cost reduction is achieved by introducing new degrees of freedom on the mesh skeleton, which are the only globally coupled portion of the scheme. We also use the log-density formulation on the PNP equations as proposed by Metti et al. (2016) [17] and also utilized in [4]. The log-density formulation is what assures positivity preservation for the density of charged particles. Finally, we also utilize a time adaptation method for efficiently computing steady-state solution problems. The energy stability of our numerical scheme is also approved. To the best of the author’s knowledge, our work is the first attempt to combine HDG and log-density formulation for solving highly nonlinear PNP equations, in which the density value could be close to zero. It is also the first time that the energy stability analysis of this type of implicit time-stepping HDG scheme for PNP equations was performed.

This paper is structured as follows. In Section 2, we introduce the PNP system of equations, and describe in detail the proposed HDG method. We finish the section by proving the system is positivity-preserving, mass-conserving, and energy-dissipative. In Section 3, we provide four distinct numerical examples. These examples cover an accuracy test, varying orders of time discretization, and one-dimensional and two-dimensional domains. In Appendix A, we derive a scheme utilizing DG temporal discretization in conjunction with the HDG spatial discretization, resulting in a HDG-DG space–time numerical scheme. In Section 4, we conclude the paper with final thoughts.

## 2. PNP System of Equations and the Hybridizable Discontinuous Galerkin Method

### 2.1. The PNP System of Equations

We begin by introducing the classical PNP system. Here we consider *N* species of charge particles on a bounded domain $\Omega \subset R^{d}$. The Poisson equation models how charge creates potential and is described below:

(1) $$-\nabla \left(\varepsilon \nabla \phi \right)=\rho_{0}+\sum_{i=1}^{N}z_{i}ec_{i}\;.$$

In Equation (1), ϕ is the electrostatic potential, *e* is the electrical unit charge, and $c_{i}$ is the concentration of the *i*-th charged particle. ε is the electric permittivity, $\rho_{0}$ is the permanent fixed charge density of the system, and $z_{i}$ is the valence.

The Nernst–Planck equation is the species transport portion of the system and is based on conservation laws. It is described below:

(2) $$\frac{\partial c_{i}}{\partial t}+\nabla \cdot \left(J_{i}\right)=0\;,\;i=1,2,\cdots ,N\;.$$

In Equation (2), $J_{i}$ is the flux that takes the following form:

(3) $$J_{i}=-D_{i}c_{i}\nabla \mu_{i}\;,\;i=1,2,\cdots N\;,$$

where $D_{i}$ is the diffusion constant, and $\mu_{i}$, denoted as the chemical potential, is of the following form:

(4) $$\mu_{i}=\log\left(c_{i}\right)+\frac{z_{i}e}{k_{B}T}\phi \equiv u_{i}+\frac{z_{i}e}{k_{B}T}\phi \;,\;i=1,2,\cdots ,N\;.$$

Given $\mu_{i}$, the flux $J_{i}$ can be rewritten as

(5) $$J_{i}=-D_{i}c_{i}\left(\nabla u_{i}+\frac{z_{i}e}{k_{B}T}\nabla \phi \right)\;,\;i=1,2,\cdots ,N\;.$$

The remaining physical parameters are defined as follows: $k_{B}$ is the Boltzmann constant, and *T* is the absolute temperature. Equations (1) and (2) make up the PNP system of equations. We note that the PNP equations discussed in the paper are non-dimensional. The dimensionless form of the PNP equations can be obtained using the non-dimensionalization process discussed in [18].

Equation (4) implies that $u_{i}=\log\left(c_{i}\right)$, which ensures $c_{i}=\exp\left(u_{i}\right)>0$ for all $u_{i}$. Taking the logarithm of the concentration is a way to preserve positivity in our system and is a tactic commonly referred to as the log-density formulation [17]. Throughout the rest of this paper, our unknowns are ϕ and $u_{i}$, where they represent the electrostatic potential and the log-density of the *i*-th particle, respectively.

Rewriting Equations (1) and (2) using the log-density notation and imposing appropriate initial and boundary conditions provides us with the final PNP system of equations of the following form:

(6a) $$\begin{array}{cc} \; & \frac{\partial \exp(u_{i})}{\partial t}=\nabla \cdot \left(D_{i}\exp\left(u_{i}\right)\left(\nabla u_{i}+\frac{z_{i}e}{k_{B}T}\nabla \phi \right)\right)\;,\;i=1,2,\cdots ,N\;, \end{array}$$

(6b) $$\begin{array}{cc} \; & -\nabla \cdot \left(\varepsilon \nabla \phi \right)=\rho_{0}+\sum_{i=1}^{N}z_{i}e\exp\left(u_{i}\right)\;, \end{array}$$

(6c) $$\begin{array}{cc} \;u_{i}\left(t=0,x\right)=\log\left(c_{i}^{0}\left(x\right)\right)\;, & \;\mathrm{on}\;\Omega \;, \end{array}$$

(6d) $$\begin{array}{cc} \; & \frac{\partial u_{i}}{\partial n}=0\;,\;\mathrm{on}\;\partial \Omega \;, \end{array}$$

(6e) $$\begin{array}{cc} \; & \frac{\partial \mu_{i}}{\partial n}=\frac{\partial \phi }{\partial n}=0\;,\;\mathrm{on}\;\partial \Omega \;, \end{array}$$

where $\frac{\partial }{\partial n}$ is the normal derivative.

### 2.2. The HDG Spatial Discretization

We let $T_{h}$ be a shape regular discretization of our domain $\Omega \subset R^{d}$, where $T_{h}=\left\{K_{i},\;i=1,...,N_{T_{h}}\right\}$ in which $K_{i}$ are shape-regular simplexes. Therefore, we state

$$\Omega =\overset{N_{T_{h}}}{\underset{i=1}{⋃}}K_{i}\;.$$

Say there are two cells that are part of the collection $T_{h}$, $K^{+}$ and $K^{-}$. We define an interior face of these cells $K^{+}$ and $K^{-}$ to be $e=\partial K^{+}\cap \partial K^{-}$, when $\partial K^{+}\cap \partial K^{-}\neq \emptyset$. An exterior, or boundary, face is defined as e=∂K∩∂Ω where *K* is a cell of $T_{h}$. We furthermore assign $E_{h}^{\circ }$ to be the set of interior faces of $T_{h}$ and $E_{h}^{\partial }$ to be the set of boundary faces of $T_{h}$. Therefore, we let $E_{h}=E_{h}^{\circ }\cup E_{h}^{\partial }$ and also define $\partial T_{h}=\left\{\partial K\;:\;K\in T_{h}\right\}$.

We now define the finite-dimensional polynomial approximation spaces to be used. For $K\in T_{h}$, we let $V_{h}$ be the polynomial space that will approximate flux values, $W_{h}$ be the space that will approximate scalar solutions, and $M_{h}$ be the space that will approximate trace values on the faces.

$$\begin{array}{c} V_{h}=\left\{v\in {\left(L^{2}\left(\Omega \right)\right)}^{d}:v{|}_{K}\in {\left(P_{p}\left(K\right)\right)}^{d},\forall K\in T_{h}\right\}\;,\\ W_{h}=\left\{w\in L^{2}\left(\Omega \right):w{|}_{K}\in P_{p}\left(K\right),\forall K\in T_{h}\right\}\;,\\ M_{h}=\left\{\mu \in L^{2}\left(E_{h}\right):\mu {|}_{F}\in P_{p}\left(F\right),\forall F\in E_{h}\right\}\;. \end{array}$$

where $P_{p}\left(K\right)$ is the space of polynomials of degree at most *p* on cell *K*, and *F* are the edges in $E_{h}$. We next introduce the standard inner product notation, commonly used by Cockburn et al. (2009) and Nguyen et al. (2011) [20,21], to simplify the description of our HDG scheme. For function $u\in L^{2}\left(\Omega \right)$ and $v\in L^{2}\left(\Omega \right)$, we write the integral of the inner product as

$${\left(u,v\right)}_{\Omega }=\int_{\Omega }uv\;dx\;,$$

where Ω is a domain in $R^{d}$. If Ω is a domain in $R^{d-1}$, we write the inner product as

$${\langle u,v\rangle }_{\Omega }=\int_{\Omega }uv\;dx\;.$$

If $w\in {\left(L^{2}\left(\Omega \right)\right)}^{d}$ and $v\in {\left(L^{2}\left(\Omega \right)\right)}^{d}$ for Ω in $R^{d}$, we let

$${\left(w,v\right)}_{\Omega }=\int_{\Omega }w\cdot vdx\;.$$

Furthermore, we adopt the standard notation that simplifies the heavily mesh-dependent integrals in the HDG scheme, also introduced in [20,21].

$${\langle \mu ,\lambda \rangle }_{E_{h}}=\sum_{F\in E_{h}}{\langle \mu ,\lambda \rangle }_{F}\;,\;{\left(w,v\right)}_{T_{h}}=\sum_{K\in T_{h}}{\left(w,v\right)}_{K}\;,\;{\langle \xi ,\rho \rangle }_{\partial T_{h}}=\sum_{K\in T_{h}}{\langle w,v\rangle }_{\partial K}\;,$$

where functions μ,λ are defined on $E_{h}$, w,v is defined on $T_{h}$, and ξ,ρ are defined on $\partial T_{h}$.

To begin deriving the HDG scheme, we introduce the following auxiliary variables:

(7) $$\begin{array}{cc} \; & L=-\nabla \phi \;,\\ \; & \omega_{i}=-D_{i}\exp\left(u_{i}\right)\left(\nabla u_{i}+\frac{z_{i}e}{k_{B}T}\nabla \phi \right)\;. \end{array}$$

We input these auxiliary variables into Equations (6a) and (6b) to obtain the following first-order formulation of the PNP model:

(8a) $$\begin{array}{cc} \; & \omega_{i}+D_{i}\exp\left(u_{i}\right)\left(\nabla u_{i}+\frac{z_{i}e}{k_{B}T}\nabla \phi \right)=0\;, \end{array}$$

(8b) $$\begin{array}{cc} \; & L+\nabla \phi =0\;, \end{array}$$

(8c) $$\begin{array}{cc} \; & \frac{\partial \exp(u_{i})}{\partial t}+\nabla \cdot \omega_{i}=0\;, \end{array}$$

(8d) $$\begin{array}{cc} \; & \nabla \cdot \left(\varepsilon L\right)=\rho_{0}+\sum_{i=1}^{N}z_{i}e\exp\left(u_{i}\right)\;. \end{array}$$

We formally define $k_{i}\left(u_{i}\right)=D_{i}\exp\left(u_{i}\right)$. The semi-discrete hybridizable discontinuous Galerkin scheme for the system of Equations (8a)–(8d) can formally be written as follows: for i=1,2,⋯,N, we seek an approximation $\left(\omega_{i,h},L_{h},u_{i,h},\phi_{h},{\hat{u}}_{i,h},{\hat{\phi }}_{h}\right)\in V_{h}\times V_{h}\times W_{h}\times W_{h}\times M_{h}\times M_{h}$, such that for t>0,

(9a) $$\begin{array}{c} {\left(k_{i}^{-1}\omega_{i,h},b_{i}\right)}_{T_{h}}-{\left(\left(u_{i,h}+\frac{z_{i}e}{k_{B}T}\phi_{h}\right),\nabla \cdot b_{i}\right)}_{T_{h}}+{\langle {\hat{u}}_{i,h}+\frac{z_{i}e}{k_{B}T}{\hat{\phi }}_{h},b_{i}\cdot n\rangle }_{\partial T_{h}}=0\;,\\ \forall b_{i}\in V_{h}\;, \end{array}$$

(9b) $$\begin{array}{cc} & {\left(L_{h},G\right)}_{T_{h}}-{\left(\phi_{h},\nabla \cdot G\right)}_{T_{h}}+{\langle {\hat{\phi }}_{h},G\cdot n\rangle }_{\partial T_{h}}=0\;,\;\forall G\in V_{h}\;, \end{array}$$

(9c) $$\begin{array}{c} {\left(\frac{\partial \exp(u_{i,h})}{\partial t},\psi_{i}\right)}_{T_{h}}-{\left(\omega_{i,h},\nabla \psi_{i}\right)}_{T_{h}}+{\langle {\hat{\omega }}_{i,h}\cdot n,\psi_{i}-{\hat{\psi }}_{i}\rangle }_{\partial T_{h}}=0\;,\\ \forall \psi_{i}\in W_{h},\;\forall \hat{\psi_{i}}\in M_{h}\;, \end{array}$$

(9d) $$\begin{array}{c} -{\left(\varepsilon L_{h},\nabla v\right)}_{T_{h}}+{\langle \varepsilon {\hat{L}}_{h}\cdot n,v-\hat{v}\rangle }_{\partial T_{h}}-{\left(\rho_{0}+\sum_{i=1}^{N}z_{i}e\exp\left(u_{i,h}\right),v\right)}_{T_{h}}=0\;,\\ \forall v\in W_{h},\;\forall \hat{v}\in M_{h}\;. \end{array}$$

The numerical fluxes in Equations (9c) and (9d) are chosen to take the following form:

$$\begin{array}{c} {\hat{\omega }}_{i,h}\cdot n=\omega_{i,h}\cdot n+\eta \alpha_{i}\left(u_{i,h}-{\hat{u}}_{i,h}\right)+\eta \xi_{i}\left(\phi_{h}-{\hat{\phi }}_{h}\right)\;,\\ \varepsilon {\hat{L}}_{h}\cdot n=\varepsilon L_{h}\cdot n+\eta \varepsilon \left(\phi_{h}-{\hat{\phi }}_{h}\right)\;, \end{array}$$

where η, $\alpha_{i}$, and  $\xi_{i}$ are the HDG stabilization parameters.

### 2.3. Positivity-Preserving, Mass-Conserving, and Energy Dissipation Properties of the Semi-Discrete HDG Scheme

The PNP system of Equation (Section 2.1) can be shown to be mass-conserving, positivity-preserving, and energy-dissipative [4]. The mass conservation property is stated as follows:

$$\begin{array}{cc} \; & \int_{\Omega }c_{i}\left(x,t\right)\;dx=\int_{\Omega }c_{i}\left(x,0\right)\;dx\;. \end{array}$$

The positivity-preserving states are given as follows:

If at t=0, $c_{i}^{0}\left(x\right)>0$, then $c_{i}\left(x,t\right)>0$ for all t>0.

The energy dissipation identity is derived using the standard energy variational argument [4], and is given by

$$\begin{array}{c} \frac{d}{dt}\int_{\Omega }\left(\sum_{i=1}^{N}\left(\exp\left(u_{i}\right)\left(u_{i}-1\right)\right)+\frac{\varepsilon }{2k_{B}T}{\left|\nabla \phi \right|}^{2}\right)\;dx=\\ -\sum_{i=1}^{N}\int_{\Omega }D_{i}\exp\left(u_{i}\right)|\nabla u_{i}+\frac{z_{i}e}{k_{B}T}\nabla \phi {|}^{2}\;dx\;, \end{array}$$

where the free energy total is the following term:

$$\int_{\Omega }\left(\sum_{i=1}^{N}\left(\exp\left(u_{i}\right)\left(u_{i}-1\right)\right)+\frac{\varepsilon }{2k_{B}T}{\left|\nabla \phi \right|}^{2}\right)\;dx\;.$$

**Theorem** **1.** *Assume $c_{i}^{0}>0$ for all i. The numerical solution to the semi-discrete scheme (Section 2.2) is mass-conserving, positivity-preserving, and energy-dissipative. The energy dissipation property is given by Equation (15).*

**Proof.**  Taking $\psi_{i}=1$ and $\hat{\psi_{i}}=1$ in Equation (9c), we immediately have the mass conservation. The positivity of numerical $c_{i}$ follows from $c_{i}=\exp\left(u_{i,h}\right)>0$.

We set out to prove the energy dissipation property of our derived semi-discrete HDG scheme. For Equation (9b), let $G=\frac{z_{i}e}{k_{B}T}\omega_{i,h}$. This gives us

(10) $$\begin{array}{cc} \; & \sum_{i=1}^{N}\sum_{K\in T_{h}}\left[{\left(L_{h},\frac{z_{i}e}{k_{B}T}\omega_{i,h}\right)}_{K}-{\left(\phi_{h},\frac{z_{i}e}{k_{B}T}\nabla \cdot \omega_{i,h}\right)}_{K}+{\langle {\hat{\phi }}_{h},\frac{z_{i}e}{k_{B}T}\omega_{i,h}\cdot n\rangle }_{\partial K}\right]=0\;. \end{array}$$

For Equation (9c), we let $\psi_{i}=\mu_{i,h}$, where $\mu_{i,h}=u_{i,h}+\frac{z_{i}e}{k_{B}T}\phi_{h}$. We also let ${\hat{\psi }}_{i}={\hat{\mu }}_{i,h}$, where ${\hat{\mu }}_{i,h}={\hat{u}}_{i,h}+\frac{z_{i}e}{k_{B}T}{\hat{\phi }}_{h}$. After substituting these terms, we integrate by parts to obtain

$$\begin{array}{cc} \sum_{i=1}^{N}\sum_{K\in T_{h}}\left[{\left(\frac{\partial \exp(u_{i,h})}{\partial t},\mu_{i,h}\right)}_{K}-{\left(\omega_{i,h},\nabla \mu_{i,h}\right)}_{K}+{\langle {\hat{\omega }}_{i,h}\cdot n,\mu_{i,h}-{\hat{\mu }}_{i,h}\rangle }_{\partial K}\right]=0\;, & \end{array}$$

$$\begin{array}{c} \sum_{i=1}^{N}\sum_{K\in T_{h}}\left[{\left(\frac{\partial \exp(u_{i,h})}{\partial t},\mu_{i,h}\right)}_{K}-{\langle \omega_{i,h}\cdot n,\mu_{i,h}\rangle }_{\partial K}+{\left(\nabla \cdot \omega_{i,h},\mu_{i,h}\right)}_{K}\right.\\ +{\langle {\hat{\omega }}_{i,h}\cdot n,\mu_{i,h}-{\hat{\mu }}_{i,h}\rangle }_{\partial K}]=0\;. \end{array}$$

We now insert the numerical flux form for ${\hat{\omega }}_{i,h}\cdot n$ as follows:

$$\begin{array}{c} \sum_{i=1}^{N}\sum_{K\in T_{h}}\left[{\left(\frac{\partial \exp(u_{i,h})}{\partial t},\mu_{i,h}\right)}_{K}-{\langle \omega_{i,h}\cdot n,\mu_{i,h}\rangle }_{\partial K}+{\left(\nabla \cdot \omega_{i,h},\mu_{i,h}\right)}_{K}\right.\\ \left.+{\langle \omega_{i,h}\cdot n,\mu_{i,h}-{\hat{\mu }}_{i,h}\rangle }_{\partial K}+{\langle \eta \left(u_{i,h}-{\hat{u}}_{i,h}+\frac{z_{i}e}{k_{B}T}\left(\phi_{h}-{\hat{\phi }}_{h}\right)\right),\mu_{i,h}-{\hat{\mu }}_{i,h}\rangle }_{\partial K}\right]=0\;. \end{array}$$

Now we insert the form for $\mu_{i,h}$ and ${\hat{\mu }}_{i,h}$ as follows:

$$\begin{array}{c} \sum_{i=1}^{N}\sum_{K\in T_{h}}\left[{\left(\frac{\partial \exp(u_{i,h})}{\partial t},u_{i,h}+\frac{z_{i}e}{k_{B}T}\phi_{h}\right)}_{K}\underline{+{\left(\nabla \cdot \omega_{i,h},u_{i,h}+\frac{z_{i}e}{k_{B}T}\phi_{h}\right)}_{K}}\right.\\ \left.\underline{-{\langle \omega_{i,h}\cdot n,{\hat{u}}_{i,h}+\frac{z_{i}e}{k_{B}T}{\hat{\phi }}_{h}\rangle }_{\partial K}}+\int_{\partial K}\eta {\left(u_{i,h}-{\hat{u}}_{i,h}+\frac{z_{i}e}{k_{B}T}\left(\phi_{h}-{\hat{\phi }}_{h}\right)\right)}^{2}\;ds\right]=0\;. \end{array}$$

We use Equation (9a) by letting $b_{i}=\omega_{i,h}$ to substitute the underlined terms from above as follows:

$$\begin{array}{c} \sum_{i=1}^{N}\sum_{K\in T_{h}}\left[{\left(\frac{\partial \exp(u_{i,h})}{\partial t},u_{i,h}+\frac{z_{i}e}{k_{B}T}\phi_{h}\right)}_{K}+{\left(k_{i}^{-1}\left(u_{i,h}\right)\omega_{i,h},\omega_{i,h}\right)}_{K}\right.\\ \left.+\int_{\partial K}\eta {\left(u_{i,h}-{\hat{u}}_{i,h}+\frac{z_{i}e}{k_{B}T}\left(\phi_{h}-{\hat{\phi }}_{h}\right)\right)}^{2}\;ds\right]=0\;. \end{array}$$

Rewriting the equation above gives us

(11) $$\begin{array}{c} \sum_{i=1}^{N}\sum_{K\in T_{h}}{\left(\frac{\partial \exp(u_{i,h})}{\partial t},u_{i,h}+\frac{z_{i}e}{k_{B}T}\phi_{h}\right)}_{K}=\sum_{i=1}^{N}\sum_{K\in T_{h}}\left[-{\left(k_{i}^{-1}\left(u_{i,h}\right)\omega_{i,h},\omega_{i,h}\right)}_{K}\right.\\ \left.-\int_{\partial K}\eta {\left(u_{i,h}-{\hat{u}}_{i,h}+\frac{z_{i}e}{k_{B}T}\left(\phi_{h}-{\hat{\phi }}_{h}\right)\right)}^{2}\;ds\right]\;. \end{array}$$

Taking advantage of our log-density formulation, we define a new variable $U_{i}$ where

$$\begin{array}{cc} \; & u_{i,h}=U_{i}^{\prime }\left(c_{i,h}\right)=\log\left(c_{i,h}\right)\\ \Rightarrow & U_{i}\left(u_{i,h}\right)\equiv \exp\left(u_{i,h}\right)\left(u_{i,h}-1\right) \end{array}$$

We use this new variable $U(u_{i,h})$ to rewrite Equation (11) into

(12) $$\begin{array}{c} \sum_{i=1}^{N}\sum_{K\in T_{h}}\left[\frac{d}{dt}\int_{K}U_{i}\left(u_{i,h}\right)\;dx+\int_{K}\frac{z_{i}e}{k_{B}T}\frac{\partial \exp(u_{i,h})}{\partial t}\phi_{h}\;dx\right]=\\ \sum_{i=1}^{N}\sum_{K\in T_{h}}\left[-{\left(k_{i}^{-1}\left(u_{i,h}\right)\omega_{i,h},\omega_{i,h}\right)}_{K}-\int_{\partial K}\eta {\left(u_{i,h}-{\hat{u}}_{i,h}+\frac{z_{i}e}{k_{B}T}\left(\phi_{h}-{\hat{\phi }}_{h}\right)\right)}^{2}\;ds\right]\;. \end{array}$$

Finally, we let $v=\frac{\partial \phi }{\partial t}/k_{B}T$ and $\hat{v}=\frac{\partial \hat{\phi }}{\partial t}/k_{B}T$ in Equation (9d) and also let $G=\frac{\varepsilon }{k_{B}T}\frac{\partial L_{h}}{\partial t}$ in Equation (9b). We also let $G=\frac{\varepsilon }{k_{B}T}L_{h}$ in Equation (9b) and take the time derivative. We then subtract these results from Equation (12). In this manner we obtain

(13) $$\begin{array}{c} \frac{d}{dt}\sum_{i=1}^{N}\sum_{K\in T_{h}}\left[\int_{K}U_{i}\left(u_{i,h}\right)\;dx+\int_{K}\frac{z_{i}e}{k_{B}T}\exp\left(u_{i,h}\right)\phi_{h}\;dx\right]+\\ \frac{d}{dt}\sum_{K\in T_{h}}\left[-\int_{K}\frac{\varepsilon }{2k_{B}T}{\left|L_{h}\right|}^{2}\;dx-\int_{\partial K}\frac{\eta \varepsilon }{2k_{B}T}{\left(\phi_{h}-{\hat{\phi }}_{h}\right)}^{2}\;ds+\int_{K}\frac{\rho_{0}}{k_{B}T}\phi_{h}\;dx\right]=\\ \sum_{i=1}^{N}\sum_{K\in T_{h}}\left[-\int_{K}k_{i}^{-1}\left(u_{i,h}\right){\left|\omega_{i,h}\right|}^{2}\;dx\right.\\ \left.-\int_{\partial K}\eta {\left(u_{i,h}-{\hat{u}}_{i,h}+\frac{z_{i}e}{k_{B}T}\left(\phi_{h}-{\hat{\phi }}_{h}\right)\right)}^{2}\;ds\right]\;. \end{array}$$

As a final step, we let $v=\frac{\phi_{h}}{k_{B}T}$ and $\hat{v}=\hat{\phi }/k_{B}T$ in Equation (9d) and let $G=\frac{\varepsilon }{k_{B}T}L_{h}$ in Equation (9b) and integrate by parts. Adding them together results in

$$\begin{array}{c} \sum_{K\in T_{h}}\left[{\left(\frac{\varepsilon }{k_{B}T}L_{h},L_{h}\right)}_{K}+\int_{\partial K}\frac{\eta \varepsilon }{k_{B}T}{\left(\phi_{h}-{\hat{\phi }}_{h}\right)}^{2}\;ds-\int_{K}\frac{\rho_{0}}{k_{B}T}\phi_{h}\;dx\right.\\ \left.-\sum_{i=1}^{N}\int_{K}\frac{z_{i}e}{k_{B}T}\exp\left(u_{i,h}\right)\phi_{h}\;dx\right]=0\;. \end{array}$$

(14) $$\begin{array}{c} \Rightarrow \sum_{i=1}^{N}\sum_{K\in T_{h}}\int_{K}\frac{z_{i}e}{k_{B}T}\exp\left(u_{i,h}\right)\phi_{h}\;dx=\sum_{K\in T_{h}}\left[\int_{K}\frac{\varepsilon }{k_{B}T}{\left|L_{h}\right|}^{2}\;dx\right.\\ \left.+\int_{\partial K}\frac{\eta \varepsilon }{k_{B}T}{\left(\phi_{h}-{\hat{\phi }}_{h}\right)}^{2}\;ds-\int_{K}\frac{\rho_{0}}{k_{B}T}\phi_{h}\;dx\right]\;. \end{array}$$

We substitute Equation (14) into Equation (13) to obtain the final energy dissipation property for the semi-discrete HDG scheme as follows:

(15) $$\begin{array}{c} \frac{d}{dt}\sum_{K\in T_{h}}\left[\sum_{i=1}^{N}\int_{K}U_{i}\left(u_{i,h}\right)\;dx+\int_{K}\frac{\varepsilon }{2k_{B}T}{\left|L_{h}\right|}^{2}\;dx\right]=\\ \sum_{i=1}^{N}\sum_{K\in T_{h}}\left[-\int_{K}k_{i}^{-1}\left(u_{i,h}\right){\left|\omega_{i,h}\right|}^{2}\;dx\right.\\ \left.-\int_{\partial K}\eta {\left(u_{i,h}-{\hat{u}}_{i,h}+\frac{z_{i}e}{k_{B}T}\left(\phi_{h}-{\hat{\phi }}_{h}\right)\right)}^{2}\;ds-\int_{\partial K}\frac{\eta \varepsilon }{2k_{B}T}{\left(\phi_{h}-{\hat{\phi }}_{h}\right)}^{2}\;ds\right]\;. \end{array}$$

This is the completion of the energy dissipation property proof.    □

In comparison to the work completed in Fu et al. (2022) [4], the mass conservation property of our system is stronger. The study by [4] has a mass conservation that holds globally due to the finite element method that is implemented, meanwhile our HDG scheme allows us to have mass conservation on an element-wise basis. On the other hand, our scheme has more dissipation due to the approximate traces of the field variables.

### 2.4. Time Discretization and Adaptive Time-Stepping

For the time discretization, we define a partition of the time domain, $0=t_{0}<t_{1}<\cdots <t_{m}=T$, where $▵t_{j}\equiv t_{j}-t_{j-1}$.

Here we utilize the implicit Euler method for time discretization. The fully discrete scheme for solving the PNP equations is defined as follows:

for i=1,2,⋯,N, find $\left(\omega_{i,h}^{\left(j\right)},L_{h}^{\left(j\right)},u_{i,h}^{\left(j\right)},\phi_{h}^{\left(j\right)},{\hat{u}}_{i,h}^{\left(j\right)},{\hat{\phi }}_{h}^{\left(j\right)}\right)\in V_{h}\times V_{h}\times W_{h}\times W_{h}\times M_{h}\times M_{h}$ satisfying

(16a) $$\begin{array}{c} {\left(k_{i}^{-1}\left(u_{i,h}^{\left(j\right)}\right)\omega_{i,h}^{\left(j\right)},b_{i}\right)}_{T_{h}}-{\left(u_{i,h}^{\left(j\right)}+\frac{z_{i}e}{k_{B}T}\phi_{h}^{\left(j\right)},\nabla \cdot b_{i}\right)}_{T_{h}}+{\langle {\hat{u}}_{i,h}^{\left(j\right)}+\frac{z_{i}e}{k_{B}T}{\hat{\phi }}_{h}^{\left(j\right)},b_{i}\cdot n\rangle }_{\partial T_{h}}=0\;,\\ \forall b_{i}\in V_{h}\;, \end{array}$$

(16b) $$\begin{array}{cc} & {\left(L_{h}^{\left(j\right)},G\right)}_{T_{h}}-{\left(\phi_{h}^{\left(j\right)},\nabla \cdot G\right)}_{T_{h}}+{\langle {\hat{\phi }}_{h}^{\left(j\right)},G\cdot n\rangle }_{\partial T_{h}}=0\;,\;\forall G\in V_{h}\;, \end{array}$$

(16c) $$\begin{array}{c} {\left(\frac{\exp\left(u_{i,h}^{\left(j\right)}\right)-\exp\left(u_{i,h}^{\left(j-1\right)}\right)}{▵t_{j}},\psi_{i}\right)}_{T_{h}}-{\left(\omega_{i,h}^{\left(j\right)},\nabla \psi_{i}\right)}_{T_{h}}+{\langle {\hat{\omega }}_{i,h}^{\left(j\right)}\cdot n,\psi_{i}-{\hat{\psi }}_{i}\rangle }_{\partial T_{h}}=0\;,\\ \forall \psi_{i}\in W_{h},\;\forall \hat{\psi_{i}}\in M_{h}\;, \end{array}$$

(16d) $$\begin{array}{c} -{\left(\varepsilon L_{h}^{\left(j\right)},\nabla v\right)}_{T_{h}}+{\langle \varepsilon {\hat{L}}_{h}^{\left(j\right)}\cdot n,v-\hat{v}\rangle }_{\partial T_{h}}-{\left(\rho_{0}+\sum_{i=1}^{N}z_{i}e\exp\left(u_{i,h}^{\left(j\right)}\right),v\right)}_{T_{h}}=0\;,\\ \forall v\in W_{h},\;\forall \hat{v}\in M_{h}\;. \end{array}$$

In combination with the implicit Euler method, we introduce a simple but effective adaptive time-stepping strategy based on Richardson extrapolation.

We let $\Delta t_{j+1}$ be the time-step taken at time $t_{j+1}$. Using step size $\Delta t_{j+1}$, the implicit Euler method yields solution $Y_{1}^{\left(j+1\right)}$ at time $t_{j+1}$. $Y_{1}^{\left(j+1\right)}$ and can be written as

$$Y_{1}^{\left(j+1\right)}=\zeta \left(t_{j}+\Delta t_{j+1}\right)+C{\left(\Delta t_{j+1}\right)}^{2}+O\left({\left(\Delta t_{j+1}\right)}^{3}\right)$$

where ζ is the exact solution, $C{\left(\Delta t_{j+1}\right)}^{2}+O\left({\left(\Delta t_{j+1}\right)}^{3}\right)$ is the local truncation error, and *C* is an unknown. Similarly, when taking two half steps, $\frac{\Delta t_{j+1}}{2}$, to reach $t_{j+1}$ we reach solution $Y_{2}^{\left(j+1\right)}$. Solution $Y_{2}^{\left(j+1\right)}$ can then be written as

$$\begin{array}{cc} Y_{2}^{\left(j+1\right)} & =\zeta \left(t_{j}+\Delta t_{j+1}\right)+\left[C{\left(\frac{\Delta t_{j+1}}{2}\right)}^{2}+O\left({\left(\Delta t_{j+1}\right)}^{3}\right)\right]+\left[C{\left(\frac{\Delta t_{j+1}}{2}\right)}^{2}+O\left({\left(\Delta t_{j+1}\right)}^{3}\right)\right]\\ \; & =\zeta \left(t_{j}+\Delta t_{j+1}\right)+\frac{C{\left(\Delta t_{j+1}\right)}^{2}}{2}+O\left({\left(\Delta t_{j+1}\right)}^{3}\right) \end{array}$$

This means that the local relative error, $r^{j+1}$, at $t_{j+1}$ of using one step vs two half steps is

$$r^{j+1}\approx \frac{\left|Y_{1}^{\left(j+1\right)}-Y_{2}^{\left(j+1\right)}\right|}{\Delta t_{j+1}}\approx \frac{|C|\Delta t_{j+1}}{2}$$

We will use this relative local error in conjunction with a user-inputted tolerance to form this adaptive time-stepping scheme that utilizes two implicit Euler steps. A write-up of this simple adaptive time-stepping algorithm is shown in Algorithm 1.
**Algorithm 1** Adaptive Time-Stepping: Applying Implicit Euler Method For Two Steps**User-defined parameters: **$\Delta t^{0}$, $\frac{\Delta t^{0}}{2}$, $\Delta t_{\max}$$t^{\mathrm{end}}$, tolerance level $\epsilon_{1},\epsilon_{2}$1:time=02:j=03:**while** $t^{\mathrm{end}}>\mathrm{time}$ **do**4:    $\mathrm{time}\leftarrow \mathrm{time}+\Delta t^{n}$5:    Solve for $c_{1}$ at $t_{j}$ using step size $\Delta t_{j}$ and using two half steps $\frac{\Delta t_{j}}{2}$6:    Solve for $c_{2}$ at $t_{j}$ using step size $\Delta t_{j}$ and using two half steps $\frac{\Delta t_{j}}{2}$7:    Solve for ϕ at $t_{j}$ using step size $\Delta t_{j}$ and using two half steps $\frac{\Delta t_{j}}{2}$8:    Compute $r^{j}$ for $c_{1},c_{2},$ and ϕ9:    $r_{\max}\leftarrow \max\left(r_{c_{1}}^{j},r_{c_{2}}^{j},r_{\phi }^{j}\right)$10:    **if** $r_{\max}>\epsilon_{1}$ **then**11:        $\Delta t_{j}\leftarrow 0.9\left(\frac{\epsilon_{1}}{r_{\max}}\right)\Delta t_{j}$12:        j←j13:    **else if** $r_{\max}<\epsilon_{2}$ **then**14:        $\Delta t_{j+1}\leftarrow \min\left(2\Delta t_{j},\Delta t_{\max}\right)$15:        j←j+116:    **else if** $\epsilon_{2}<r_{\max}\leq \epsilon_{1}$ **then**17:        $\Delta t_{j+1}\leftarrow \Delta t_{j}$18:        j←j+119:    **end if**20:**end while**

With the implicit Euler time-stepping, we also have the following results for scheme (Section 2.4).

**Theorem** **2.** *Assume $c_{i}^{0}>0$ for all i. The numerical solution to the fully discrete scheme (Section 2.4) is mass-conserving, positivity-preserving, and energy-dissipative. The energy dissipation property is given by Equation (20).*

**Proof.**  Here we only prove the energy stability result. We use a technique introduced in [17] for this purpose. The convexity of the function f(c)=c(log(c)−1) for c>0 leads to $\left(c_{j}-c_{j-1}\right)\log c_{j}\geq c_{j}\left(\log c_{j}-1\right)-c_{j-1}\left(\log c_{j-1}-1\right)$. Applying this bound with $c_{j}=\exp\left(u_{i,h}^{\left(j\right)}\right)$ and $c_{j}=\exp\left(u_{i,h}^{\left(j-1\right)}\right)$, we obtain

(17) $$\begin{array}{c} {\left(\frac{\exp\left(u_{i,h}^{\left(j\right)}\right)-\exp\left(u_{i,h}^{\left(j-1\right)}\right)}{▵t_{j}},u_{i,h}^{\left(j\right)}\right)}_{T_{h}}\geq \;\;\;\;\;\;\;\;\;\;\;\;\;\;\;\;\;\;\;\;\;\;\;\;\;\;\;\;\\ \frac{{\left(\exp\left(u_{i,h}^{\left(j\right)}\right),u_{i,h}^{\left(j\right)}-1\right)}_{T_{h}}-{\left(\exp\left(u_{i,h}^{\left(j-1\right)}\right),u_{i,h}^{\left(j-1\right)}-1\right)}_{T_{h}}}{▵t_{j}}\;. \end{array}$$

To estimate

$$\begin{array}{c} \sum_{i=1}^{N}{\left(\frac{\exp\left(u_{i,h}^{\left(j\right)}\right)-\exp\left(u_{i,h}^{\left(j-1\right)}\right)}{▵t_{j}},\frac{z_{i}e}{k_{B}T}\phi_{h}^{\left(j\right)}\right)}_{T_{h}}\;, \end{array}$$

we subtract consecutive time-steps of Equation (9d) and utilize Equation (9b) and the Cauchy–Schwarz inequality to give rise to

(18) $$\begin{array}{c} \sum_{i=1}^{N}{\left(\frac{\exp\left(u_{i,h}^{\left(j\right)}\right)-\exp\left(u_{i,h}^{\left(j-1\right)}\right)}{▵t_{j}},\frac{z_{i}e}{k_{B}T}\phi_{h}^{\left(j\right)}\right)}_{T_{h}}\geq \frac{1}{2▵t_{j}}{\left(L_{h}^{\left(j\right)},\frac{\varepsilon }{k_{B}T}L_{h}^{\left(j\right)}\right)}_{T_{h}}\\ -\frac{1}{2▵t_{j}}{\left(L_{h}^{\left(j-1\right)},\frac{\varepsilon }{k_{B}T}L_{h}^{\left(j-1\right)}\right)}_{T_{h}}\\ +\frac{1}{2▵t_{j}}{\langle \frac{\eta \varepsilon }{k_{B}T}\left(\phi_{h}^{\left(j\right)}-{\hat{\phi }}_{h}^{\left(j\right)}\right),\left(\phi_{h}^{\left(j\right)}-{\hat{\phi }}_{h}^{\left(j\right)}\right)\rangle }_{\partial T_{h}}-\\ \frac{1}{2▵t_{j}}{\langle \frac{\eta \varepsilon }{k_{B}T}\left(\phi_{h}^{\left(j-1\right)}-{\hat{\phi }}_{h}^{\left(j-1\right)}\right),\left(\phi_{h}^{\left(j-1\right)}-{\hat{\phi }}_{h}^{\left(j-1\right)}\right)\rangle }_{\partial T_{h}}\;. \end{array}$$

We define the discrete energy functional at time level *j* by

$$E_{h}^{\left(j\right)}=\sum_{K\in T_{h}}\left[\sum_{i=1}^{N}\int_{K}U_{i}\left(u_{i,h}^{\left(j\right)}\right)\;dx+\int_{K}\frac{\varepsilon }{2k_{B}T}{\left|L_{h}^{\left(j\right)}\right|}^{2}\;dx\right]\;.$$

The rest of the proof basically follows Theorem 1. The discrete energy satisfies

(19) $$\begin{array}{c} E_{h}^{\left(j\right)}-E_{h}^{\left(j-1\right)}\leq ▵t_{j}\sum_{i=1}^{N}\sum_{K\in T_{h}}\left[-\int_{K}k_{i}^{-1}\left(u_{i,h}^{\left(j\right)}\right){\left|\omega_{i,h}^{\left(j\right)}\right|}^{2}\;dx\right.\;\;\;\;\;\;\;\;\;\;\;\;\;\;\;\;\;\;\;\;\;\;\;\;\;\;\;\;\;\;\;\\ \left.-\int_{\partial K}\eta {\left(u_{i,h}^{\left(j\right)}-{\hat{u}}_{i,h}^{\left(j\right)}+\frac{z_{i}e}{k_{B}T}\left(\phi_{h}^{\left(j\right)}-{\hat{\phi }}_{h}^{\left(j\right)}\right)\right)}^{2}\;ds\right]\\ +\sum_{i=1}^{N}\sum_{K\in T_{h}}\left[-\int_{\partial K}\frac{\eta \varepsilon }{2k_{B}T}{\left(\phi_{h}^{\left(j\right)}-{\hat{\phi }}_{h}^{\left(j\right)}\right)}^{2}\;ds+\int_{\partial K}\frac{\eta \varepsilon }{2k_{B}T}{\left(\phi_{h}^{\left(j-1\right)}-{\hat{\phi }}_{h}^{\left(j-1\right)}\right)}^{2}\;ds\right]\;. \end{array}$$

The left side of Equation (19) forms a telescoping sum; summation over *j* gives rise to

(20) $$\begin{array}{c} E_{h}^{\left(m\right)}\leq E_{h}^{\left(0\right)}-\sum_{j=1}^{m}▵t_{j}\sum_{i=1}^{N}\sum_{K\in T_{h}}\left[-\int_{K}k_{i}^{-1}\left(u_{i,h}^{\left(j\right)}\right){\left|\omega_{i,h}^{\left(j\right)}\right|}^{2}\;dx\right.\;\;\;\;\;\;\;\;\;\;\;\;\;\;\;\;\;\;\;\;\;\;\\ \left.-\int_{\partial K}\eta {\left(u_{i,h}^{\left(j\right)}-{\hat{u}}_{i,h}^{\left(j\right)}+\frac{z_{i}e}{k_{B}T}\left(\phi_{h}^{\left(j\right)}-{\hat{\phi }}_{h}^{\left(j\right)}\right)\right)}^{2}\;ds\right]\\ -\sum_{i=1}^{N}\sum_{K\in T_{h}}\int_{\partial K}\frac{\eta \varepsilon }{2k_{B}T}{\left(\phi_{h}^{\left(m\right)}-{\hat{\phi }}_{h}^{\left(m\right)}\right)}^{2}ds+\sum_{i=1}^{N}\sum_{K\in T_{h}}\int_{\partial K}\frac{\eta \varepsilon }{2k_{B}T}{\left(\phi_{h}^{\left(0\right)}-{\hat{\phi }}_{h}^{\left(0\right)}\right)}^{2}\;ds\;. \end{array}$$

Notice that the last term on the right side of Equation (20) can be made zero with an appropriate choice of project operator for initialization. This completes the proof of the energy dissipation property of the fully discrete scheme. □

## 3. Numerical Examples

We provide an assortment of examples for solving the PNP system. The simulations in this paper were run using the open-source high-performance finite element software NGSolve: https://ngsolve.org/ [22].

### 3.1. Example 1: Accuracy Test

We start with an example that has a manufactured known solution in order to test the accuracy of our scheme (Section 2.2). This known solution and test parameters are taken from [4]. We will use homogeneous Dirichlet boundary conditions on $u_{1},u_{2},$ and ϕ. By letting $N=2,z_{1}=1,z_{2}=-1,e=k_{B}=T=D_{1}=D_{2}=\varepsilon =1$, we assign the known solution to be

(21) $$\begin{array}{cc} \; & c_{1}\left(t,x,y\right)=1+0.5\sin\left(t\right)\sin\left(\pi x\right)\sin\left(\pi y\right)\;,\\ \; & c_{2}\left(t,x,y\right)=1-0.5\sin\left(t\right)\sin\left(\pi x\right)\sin\left(\pi y\right)\;,\\ \; & \phi (t,x,y)=\sin(t)\sin(\pi x)\sin(\pi y)\;. \end{array}$$

This solution introduces source terms $f_{1}$ and $f_{2}$ into our model equations, meaning the model equations can be re-written as

$$\begin{array}{cc} \; & \frac{\partial \exp(u_{1})}{\partial t}=\nabla \cdot \left(D_{1}\exp\left(u_{1}\right)\left(\nabla u_{1}+\frac{z_{1}e}{k_{B}T}\nabla \phi \right)\right)+f_{1}\;,\\ \; & \frac{\partial \exp(u_{2})}{\partial t}=\nabla \cdot \left(D_{2}\exp\left(u_{2}\right)\left(\nabla u_{2}+\frac{z_{2}e}{k_{B}T}\nabla \phi \right)\right)+f_{2}\;,\\ \; & -\nabla \cdot \left(\varepsilon \nabla \phi \right)=\rho_{0}+z_{1}e\exp\left(u_{1}\right)+z_{2}e\exp\left(u_{2}\right)\;. \end{array}$$

We choose an implicit Euler time discretization and let $\Delta t=0.1h^{k+1}$, where the element size $h=1/n_{x}$. The $L^{2}$ errors at time t=1 are shown in Table 1 along with the accompanying convergence rates. We note that the numerical solution of the HDG scheme can achieve super-convergence by cell-wise post processing [23], but this is not the focus of the current work. Nevertheless, utilizing the higher-order polynomials can improve the numerical solution resolution while reducing the number of grid cells as in standard practice of high-order schemes.

### 3.2. Example 2: One-Dimensional Domain with Implicit Euler Time Discretization

For this example, we let our one-dimensional computational domain be Ω, where Ω=[−28,25]. We also let N=2 and incorporate the cross-section area of the ion channel *A*, where $A=\pi r^{2}=\pi r{\left(x\right)}^{2}$ (assuming an axis-symmetric ion channel geometric configuration). Using homogeneous Dirichlet boundary conditions, we define our PNP model problem as

(22) $$\begin{array}{cc} \; & A\frac{\partial \exp(u_{1})}{\partial t}=\frac{\partial }{\partial x}\left(AD_{1}\exp\left(u_{1}\right)\left(\frac{\partial u_{1}}{\partial x}+\frac{z_{1}e}{k_{B}T}\frac{\partial \phi }{\partial x}\right)\right)\\ \; & A\frac{\partial \exp(u_{2})}{\partial t}=\frac{\partial }{\partial x}\left(AD_{2}\exp\left(u_{2}\right)\left(\frac{\partial u_{2}}{\partial x}+\frac{z_{2}e}{k_{B}T}\frac{\partial \phi }{\partial x}\right)\right)\\ \; & -\frac{\partial }{\partial x}\left(A\varepsilon \frac{\partial \phi }{\partial x}\right)=A\left(\rho_{0}+z_{1}e\exp\left(u_{1}\right)+z_{2}e\exp\left(u_{2}\right)\right)\\ \; & u_{1}=0\;\;\mathrm{on}\;\partial \Omega \\ \; & u_{2}=0\;\;\mathrm{on}\;\partial \Omega \\ \; & \phi =0\;\;\mathrm{on}\;\partial \Omega \end{array}$$

We let the HDG-stabilization parameters be defined as

$$\begin{array}{cc} \; & \alpha_{1}=AD_{1}\exp\left(u_{1}\right)\;,\\ \; & \alpha_{2}=AD_{2}\exp\left(u_{2}\right)\;,\\ \; & \eta =\frac{10k^{2}}{h^{2}}\;,\\ \; & \xi_{1}=\frac{z_{1}e}{k_{B}T}AD_{1}\exp\left(u_{1}\right)\;,\\ \; & \xi_{2}=\frac{z_{2}e}{k_{B}T}AD_{2}\exp\left(u_{2}\right)\;, \end{array}$$

where *k* is the polynomial order and *h* is the mesh size. The other parameter values are assigned to be

$$\begin{array}{cc} \; & k_{B}=T=e=z_{1}=D_{1}=1\;,\\ \; & z_{2}=-1\;,\\ \; & D_{2}=1.0383\;. \end{array}$$

**Figure 1:**
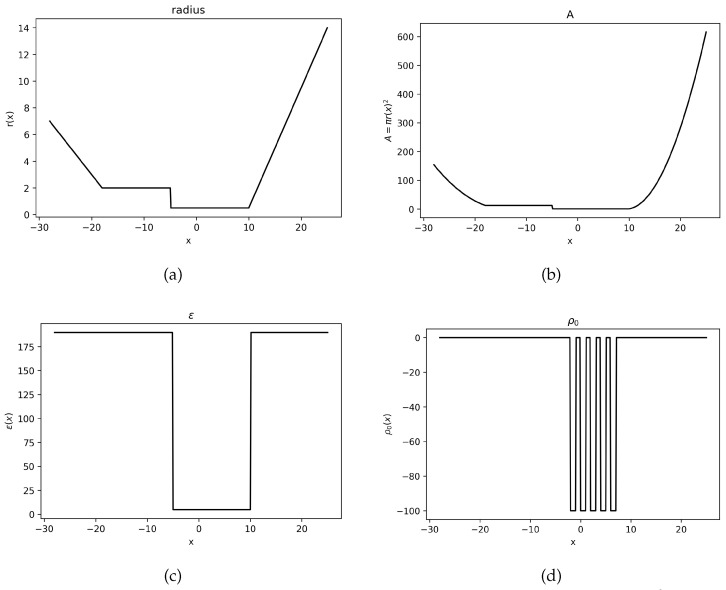
The parameter values used for **Example 2**. (**a**) Plot of r(x). (**b**) Plot of $A=\pi r{\left(x\right)}^{2}$. (**c**) Plot of ε(x). (**d**) Plot of $\rho_{0}\left(x\right)$.

The cross-section ion channel area, electric permittivity, and permanent fixed charge density take the following values, respectively. These parameter values can be seen graphically in Figure 1.

$$A=\pi r^{2},\;\mathrm{where}\;r\left(x\right)=\left\{\begin{array}{cc} -0.5x-7 & \mathrm{if}\;-28<x<-18\;,\\ 2 & \mathrm{if}\;-18<x<-5\;,\\ 0.5 & \mathrm{if}\;-5<x<10\;,\\ 0.9x-8.5 & \mathrm{if}\;10<x<25\;, \end{array}\right.$$

$$\varepsilon \left(x\right)=\left\{\begin{array}{cc} 4.7448 & \mathrm{if}-5<x<-10\;,\\ 189.79 & \mathrm{elsewhere}\;, \end{array}\right.$$

$$\rho_{0}\left(x\right)=\left\{\begin{array}{cc} -100 & \mathrm{if}\;x\in (-2,-1)\cup (0,1)\cup (2,3)\cup (4,5)\cup (6,7)\;,\\ 0 & \mathrm{elsewhere}\;. \end{array}\right.$$

**Figure 2:**
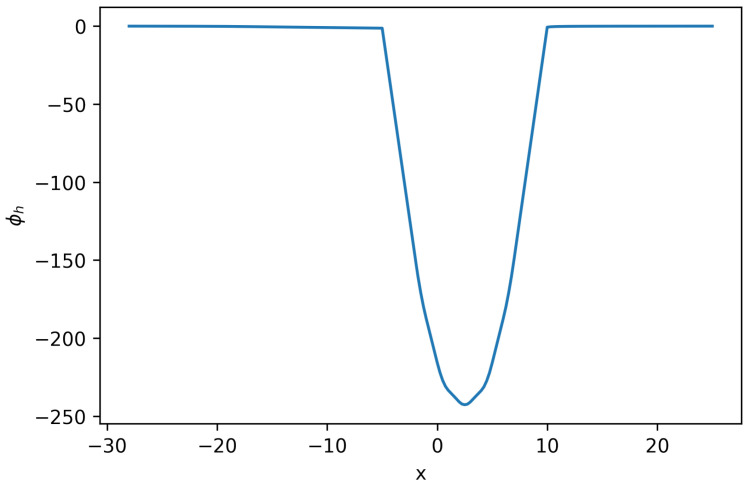
Solution $\phi_{h}$ for Equation (22) (**Example 2**).

**Figure 3:**
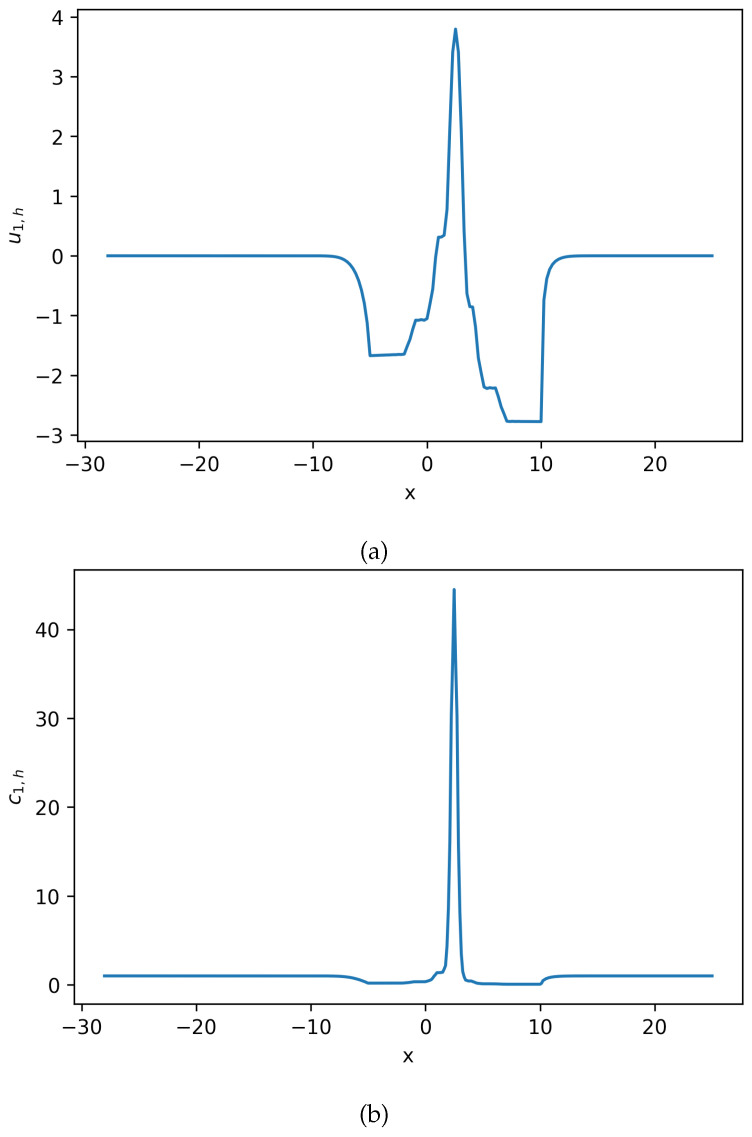
Estimated solution $u_{1,h}$ and $c_{1,h}$ for Equation (22), where $c_{1,h}=\exp\left(u_{1,h}\right)$. (**a**) Numerical solution $u_{1,h}$ for Equation (22). (**b**) Numerical solution $c_{1,h}$ for Equation (22). This density solution develops a large spike, and the solution maintains positive (**Example 2**).

**Figure 4:**
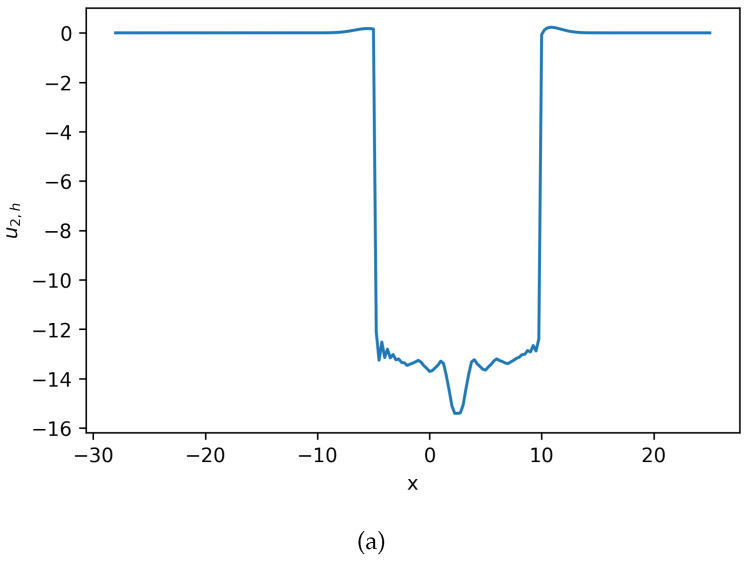

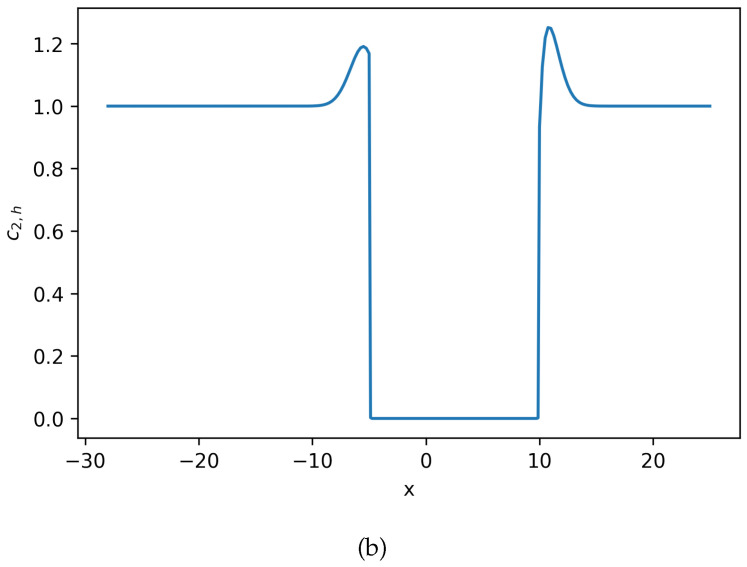
Estimated solution $u_{2,h}$ and $c_{2,h}$ for Equation (22), where $c_{2,h}=\exp\left(u_{2,h}\right)$. (**a**) Numerical solution $u_{2,h}$ for Equation (22). (**b**) Numerical solution $c_{2,h}$ for Equation (22) (**Example 2**).

Using these defined parameters values, polynomial order k=1, and Δt=0.005, the solution to the system of Equation (22) can be seen in Figure 2,Figure 3 and Figure 4.

### 3.3. Example 3: One-Dimensional Domain with Second-Order Midpoint Time Discretization

Here we show that our semi-discrete HDG scheme can also be combined with other time-stepping methods to yield a stable scheme, althrough we do not formally prove the stability of the fully discrete scheme. Similar to Example 2, our computational one-dimensional domain is Ω=[−28,25]. We solve the same model problem, Equation (22), and the same parameter values from Example 2. For the time discretization, we use the Backward Euler/Forward Euler (BEFE) scheme as described in Burkardt et al. (2020) [24]. This second-order accurate scheme is achieved by solving a Backward Euler scheme at a half time-step, to reach $t_{j+\frac{1}{2}}$. Then, a Forward Euler scheme is solved for another half time-step to reach $t_{j+1}$. For clarification, see the two half time-step equations below:

$$\begin{array}{cc} \; & \left(\mathrm{BE}\right)\;\;\frac{u_{j+\frac{1}{2}}-u_{j}}{\Delta t/2}=f\left(t_{j+\frac{1}{2}},u_{j+\frac{1}{2}}\right)\\ \; & \left(\mathrm{FE}\right)\;\;\frac{u_{j+1}-u_{j+\frac{1}{2}}}{\Delta t/2}=f\left(t_{j+\frac{1}{2}},u_{j+\frac{1}{2}}\right) \end{array}$$

**Figure 5:**
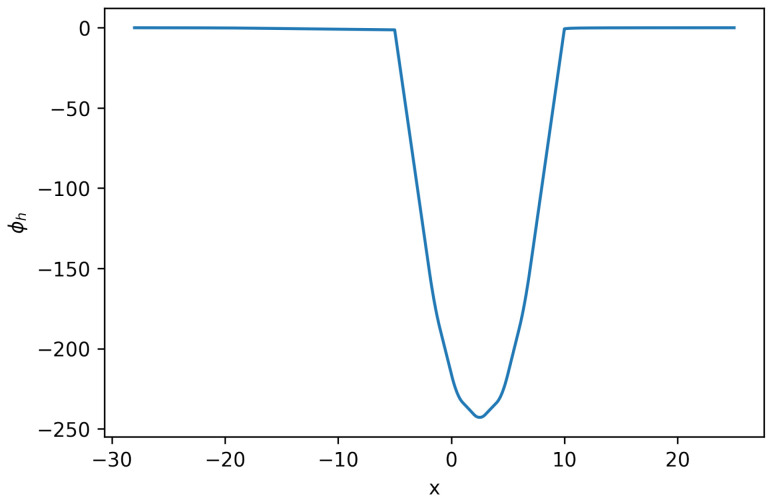
Solution $\phi_{h}$ for Equation (22) (**Example 3**). Notice that there is almost no noticeable difference between this result and result in Figure 2. The second-order time discretization does not significantly improve the solution resolution.

**Figure 6:**
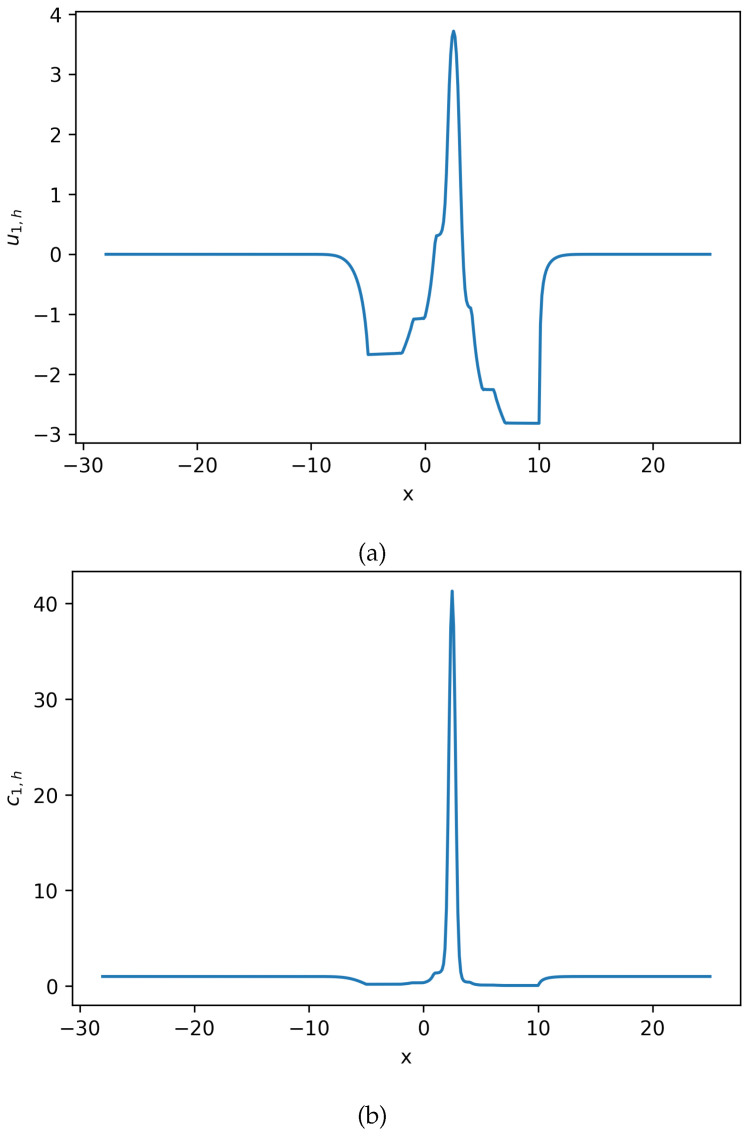
Estimated solution $u_{1,h}$ and $c_{1,h}$ for Equation (22), where $c_{1,h}=\exp\left(u_{1,h}\right)$. (**a**) Numerical solution $u_{1,h}$ for Equation (22). (**b**) Numerical solution $c_{1,h}$ for Equation (22). No negative density is observed (**Example 3**). Notice that there is slight difference between this result and result in Figure 3. The second-order time discretization does not significantly improve the solution resolution.

**Figure 7:**
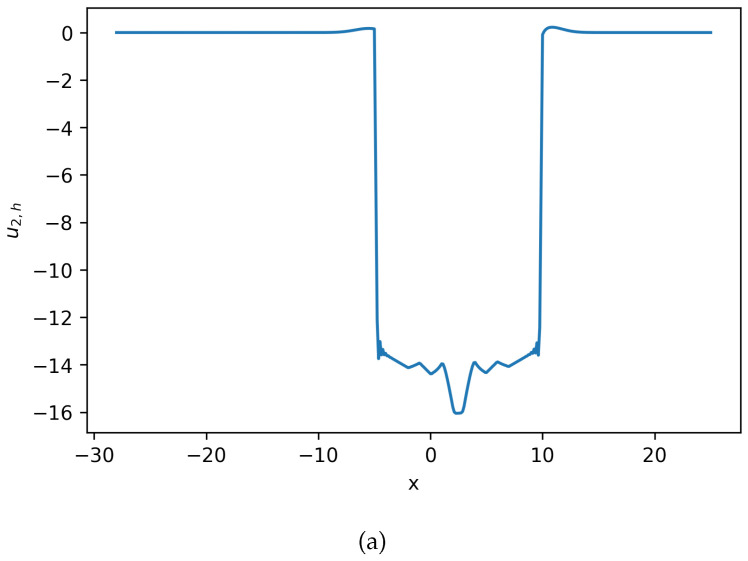

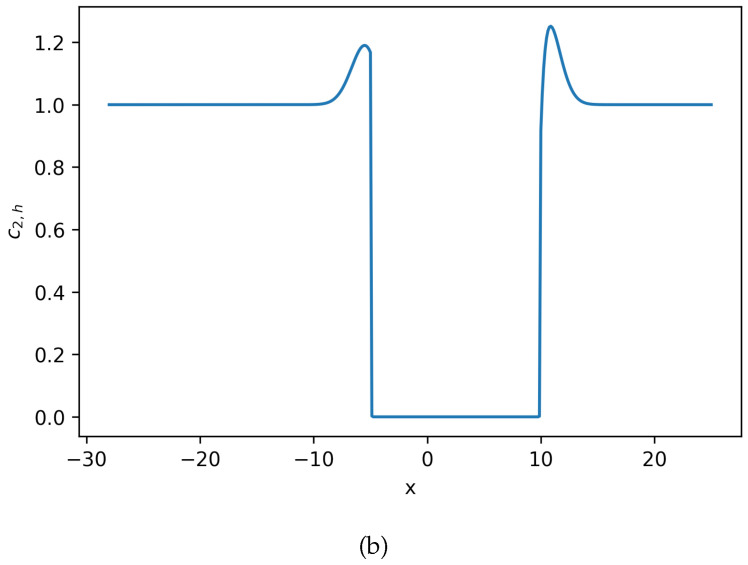
Estimated solution $u_{2,h}$ and $c_{2,h}$ for Equation (22), where $c_{2,h}=\exp\left(u_{2,h}\right)$. (**a**) Numerical solution $u_{2,h}$ for Equation (22). (**b**) Numerical solution $c_{2,h}$ for Equation (22). No negative density is observed (**Example 3**). Notice that there is slight difference between this result and result in Figure 2. The second-order time discretization does not significantly improve the solution resolution.

It can be seen that $u_{n+1}$ will be updated by $u_{j+1}=2u_{j+\frac{1}{2}}-u_{j}$. Using this BEFE time scheme, polynomial order k=2, h=0.25, and Δt=0.005, the solution of the system of Equation (22) can be seen in Figure 5,Figure 6 and Figure 7.

### 3.4. Example 4: Two-Dimensional Domain

**Figure 8:**
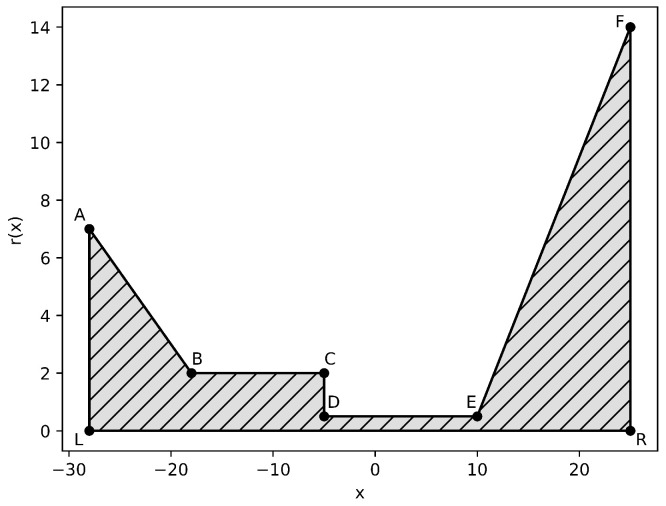
Ion Channel Geometry: Coordinates of the domain Ω are: A:(−28,7), B:(−18,2), C:(−5,2), D:(−5,0.5), E:(10,0.5), F:(25,14), L:(−28,0), R:(25,0) (**Example 4**).

We now solve the same model problem from Example 2, but let our computational domain be two-dimensional. Our domain Ω can then be defined as the channel shown in Figure 8. We let the cross-section parameter *A* take into account the new two-dimensional geometry by setting A=πr(x), where

$$r\left(x\right)=\left\{\begin{array}{cc} -0.5x-7 & \mathrm{if}\;-28<x<-18\;,\\ 2 & \mathrm{if}\;-18<x<-5\;,\\ 0.5 & \mathrm{if}\;-5<x<10\;,\\ 0.9x-8.5 & \mathrm{if}\;10<x<25\;. \end{array}\right.$$

In addition to solving on a two-dimensional domain, we will now be using homogeneous Dirichlet and homogeneous Neumann boundary conditions. Let the homogeneous Dirichlet boundary conditions be $\Gamma_{D}$, where $\Gamma_{D}=\bar{\mathrm{AL}}\cup \bar{\mathrm{FR}}$. Similarly, let the homogeneous Neumann boundary conditions be $\Gamma_{N}$, where $\Gamma_{N}=\bar{\mathrm{AB}}\cup \bar{\mathrm{BC}}\cup \bar{\mathrm{CD}}\cup \bar{\mathrm{DE}}\cup \bar{\mathrm{EF}}\cup \bar{\mathrm{LR}}$. Finally, we let $\partial \Omega =\Gamma_{D}\cup \Gamma_{N}$. After implementing these changes, our model problem for Example 4 becomes

(23) $$\begin{array}{cc} \; & A\frac{\partial \exp(u_{i})}{\partial t}=\nabla \cdot \left(AD_{i}\exp\left(u_{i}\right)\left(\nabla u_{i}+\frac{z_{i}e}{k_{B}T}\nabla \phi \right)\right)\\ \; & -\nabla \cdot \left(A\varepsilon \nabla \phi \right)=A\left(\rho_{0}+\sum_{i=1}^{N}z_{i}e\exp\left(u_{i}\right)\right)\\ \; & u_{i}=0\;\mathrm{on}\;\;\;\Gamma_{D}\\ \; & \phi =0\;\mathrm{on}\;\;\;\Gamma_{D}\\ \; & \left(-AD_{i}\exp\left(u_{i}\right)\left(\nabla u_{i}+\frac{z_{i}e}{k_{B}T}\nabla \phi \right)\right)\cdot n=0\;\;\;\;\;\;\mathrm{on}\;\;\;\Gamma_{N}\\ \; & -\left(A\varepsilon \nabla \phi \right)\cdot n=0\;\mathrm{on}\;\;\;\Gamma_{N} \end{array}$$

**Figure 9:**
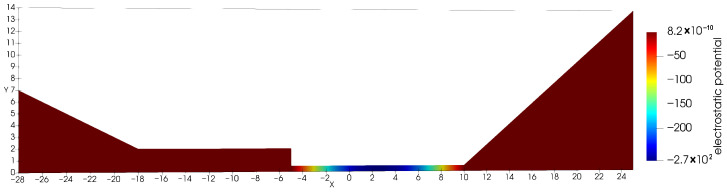
Solution $\phi_{h}$ for Equation (23) (**Example 4**).

**Figure 10:**
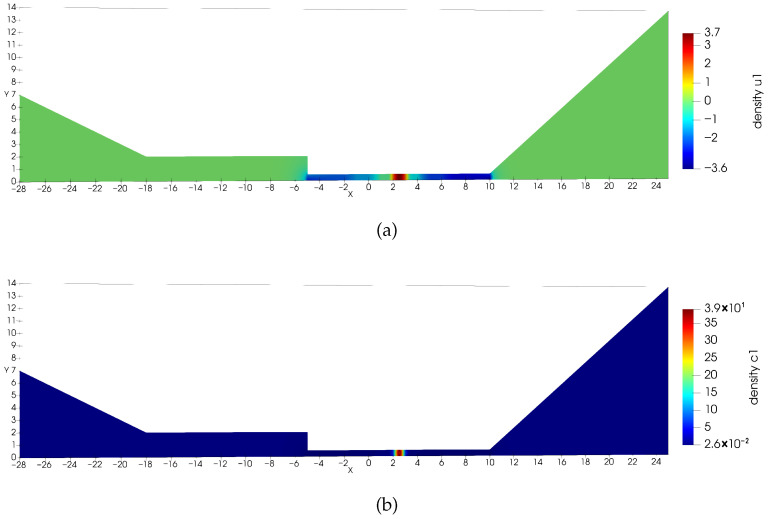
(**a**) Numerical solution $u_{1,h}$ for Equation (23) at t=1. (**b**) Numerical solution $c_{1,h}$ for Equation (23) at t=1. Positivity of density is preserved (**Example 4**).

**Figure 11:**
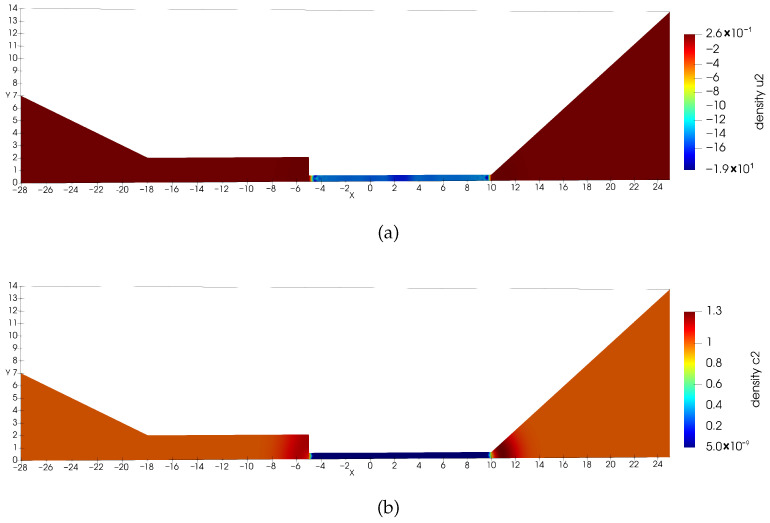
(**a**) Numerical solution $u_{2,h}$ for Equation (23) at t=1. (**b**) Numerical solution $c_{2,h}$ for Equation (23) at t=1. Positivity of density is preserved (**Example 4**).

**Figure 12:**
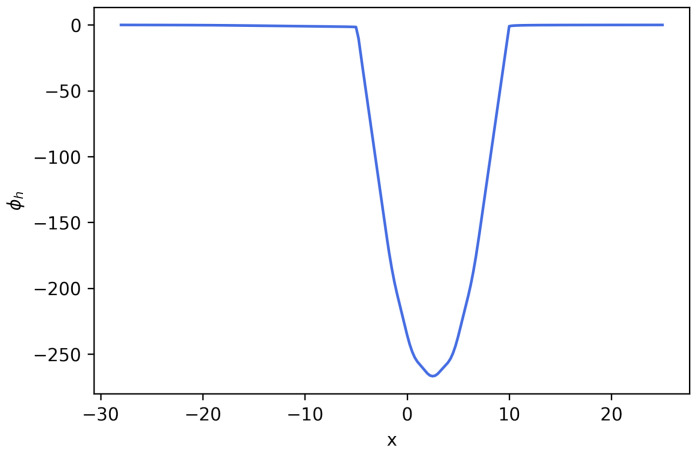
Numerical solution $\phi_{h}$ for Equation (23) cut at y=0 (**Example 4**).

**Figure 13:**
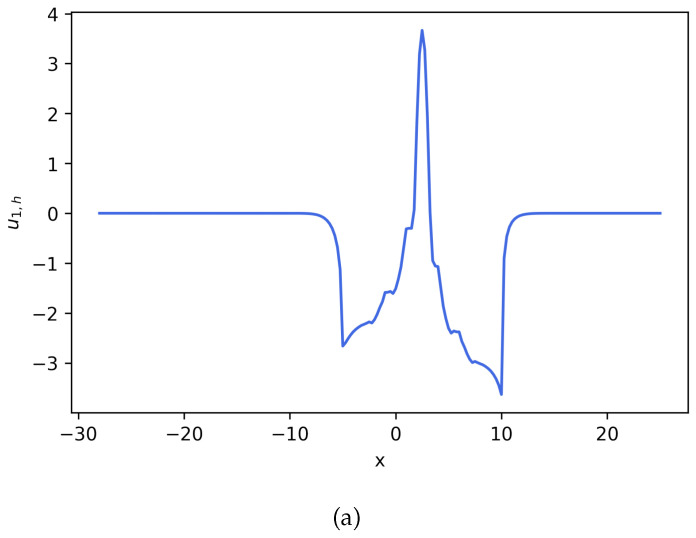

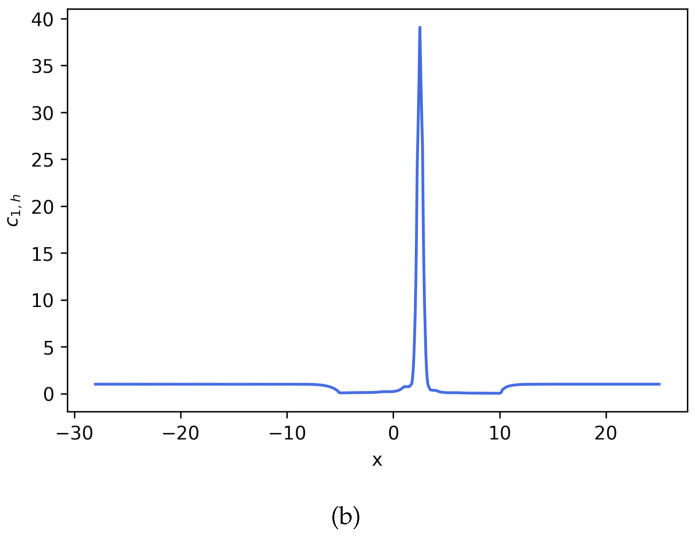
Numerical solution $u_{1,h}$ and $c_{1,h}$ for Equation (23) cut at y=0, where $c_{1,h}=\exp\left(u_{1,h}\right)$. (**a**) Numerical solution $u_{1,h}$ for Equation (23) cut at y=0. (**b**) Numerical solution $c_{1,h}$ for Equation (23) cut at y=0, where $c_{1,h}=\exp\left(u_{1,h}\right)$. The density solution develops a spike, while maintaining positivity (**Example 4**).

**Figure 14:**
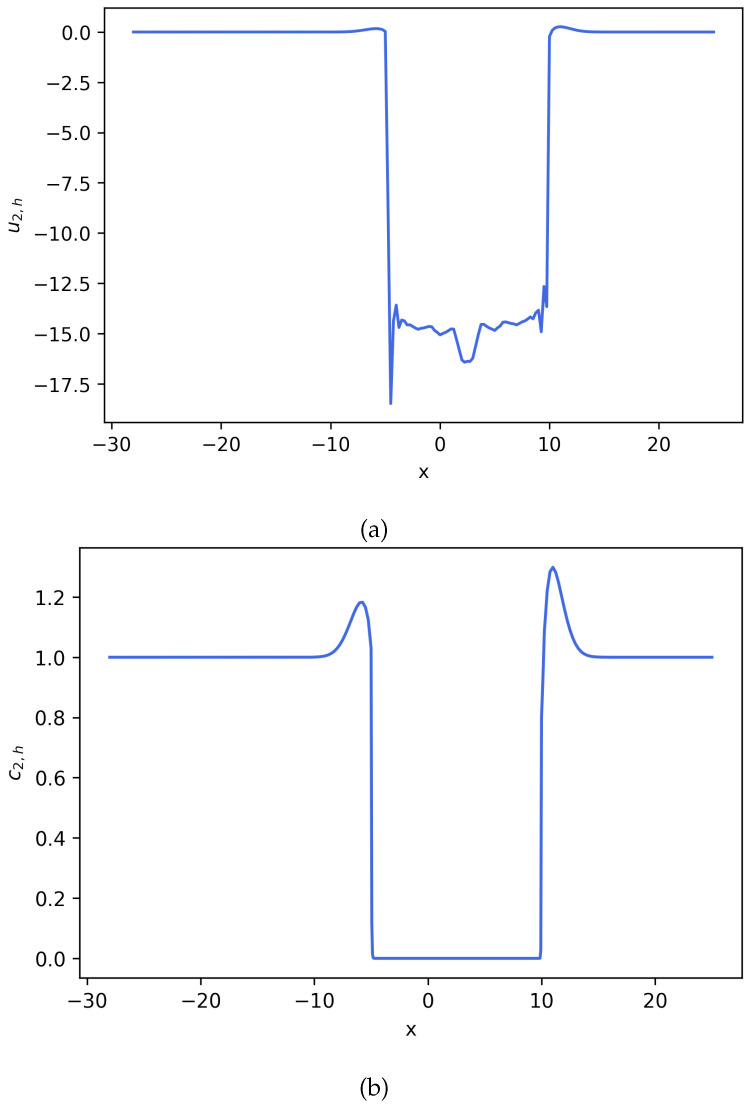
Numerical solution $u_{2,h}$ and $c_{2,h}$ for Equation (23) cut at y=0, where $c_{2,h}=\exp\left(u_{2,h}\right)$. (**a**) Numerical solution $u_{2,h}$ for Equation (23) cut at y=0. (**b**) Numerical solution $c_{2,h}$ for Equation (23) cut at y=0. The density solution is very close to zero, and still maintains positivity (**Example 4**).

We solve the system of Equation (23) using an HDG scheme for spatial discretization and an implicit Euler time-stepping scheme. The HDG stabilization parameters η, $\alpha_{i}$, and $\xi_{i}$ take the same values assigned in Example 2. The physical parameters of the problem, except for *A*, also take the same values as Example 2. Using these defined parameter values, polynomial order k=1, Δt=0.005, and h=0.25, the solution on domain Ω at t=1 can be seen in Figure 9, Figure 10 and Figure 11. To easily compare these results to solutions from the previous examples, we include Figure 12, Figure 13 and Figure 14, which contain the two-dimension solution cut at y=0.

## 4. Conclusions

In this work, we presented an HDG method implemented for solving the PNP equations. This method allows us to retain many positive properties from the DG methods, i.e., a reduction in computational cost, the ease of boundary condition implementation, and excellent performance on non-uniform meshes. The examples provided demonstrate that our scheme is accurate and preserves these desired properties.

As part of future avenues of research, we also provide the derivation of our HDG-DG space–time scheme in Appendix A; this would allow us to gain energy stability properties while achieving high-order accuracy in time. However, we leave the simulation implementation of the space–time HDG-DG scheme for future work when NGSolve supports such implementation (currently, NGSOLVE does not have a solver for using a space–time HDG-DG finite element space). We also wish to prove that our scheme is Maximum-Principle-Preserving (MPP). Schemes that uphold the MPP property are desirable because they are a way of avoiding false oscillations in the solution, especially at discontinuous locations. Techniques have been developed that, when applied to a scheme, can enforce the MPP property [25,26].

In conclusion, the HDG scheme is an excellent choice of method for solving the PNP equations since this scheme has a lower computational cost than traditional DG schemes. With biological parameter values and appropriate domain dimensions taken from the literature, this scheme could be used to model a physical ion channel.

## Figures and Tables

**Table 1:** Convergence rate table showing $L^{2}$ errors of $c_{1,h}$, $c_{2,h}$, and $\phi_{h}$, at time t=1 for scheme (Section 2.2) (**Example 1**).

Scheme Order	$n_{x}$	$L^{2}$-err in $c_{1,h}$	Rate	$L^{2}$-err in $c_{2,h}$	Rate	$L^{2}$-err in $\phi_{h}$	Rate
k=1	4	$1.539\times 10^{-2}$	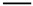	$1.054\times 10^{-2}$	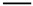	$2.868\times 10^{-2}$	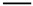
	8	$3.612\times 10^{-3}$	2.09	$2.450\times 10^{-3}$	2.11	$6.758\times 10^{-3}$	2.09
	16	$8.787\times 10^{-4}$	2.04	$5.951\times 10^{-4}$	2.04	$1.643\times 10^{-3}$	2.04
	32	$2.169\times 10^{-4}$	2.02	$1.469\times 10^{-4}$	2.02	$4.053\times 10^{-4}$	2.02
	64	$5.387\times 10^{-5}$	2.01	$3.651\times 10^{-5}$	2.01	$1.006\times 10^{-4}$	2.01
k=2	4	$3.700\times 10^{-4}$	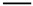	$1.023\times 10^{-3}$	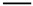	$1.445\times 10^{-3}$	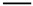
	8	$4.490\times 10^{-5}$	3.04	$1.254\times 10^{-4}$	3.03	$1.780\times 10^{-4}$	3.02
	16	$5.516\times 10^{-6}$	3.03	$1.547\times 10^{-5}$	3.02	$2.198\times 10^{-5}$	3.02
	32	$6.837\times 10^{-7}$	3.01	$1.920\times 10^{-6}$	3.01	$2.729\times 10^{-6}$	3.01
	64	$8.369\times 10^{-8}$	3.03	$2.358\times 10^{-7}$	3.03	$3.365\times 10^{-7}$	3.02
k=3	4	$4.470\times 10^{-5}$	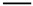	$1.041\times 10^{-4}$	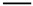	$1.065\times 10^{-4}$	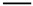
	8	$2.875\times 10^{-6}$	3.96	$6.902\times 10^{-6}$	3.91	$6.644\times 10^{-6}$	4.00
	16	$1.803\times 10^{-7}$	4.00	$4.315\times 10^{-7}$	4.00	$4.139\times 10^{-7}$	4.00
	32	$1.119\times 10^{-8}$	4.01	$2.678\times 10^{-8}$	4.01	$2.571\times 10^{-8}$	4.01
	64	$7.001\times 10^{-10}$	4.00	$1.672\times 10^{-9}$	4.00	$1.598\times 10^{-9}$	4.01

## Data Availability

Dataset (code) available on request from the authors. Interested users can go to https://github.com/NGSolve/ngsolve?tab=readme-ov-file (accessed on 28 October 2025) to follow tutorials for installing the necessary packages. The code script can run on both Windows and MacOS machines.

## Appendix A. Discontinuous Galerkin Temporal Discretization

We derive a temporal discretization of our scheme, the system of Equation (Section 2.2), by using the DG method. For this, we let $t_{n}$ be the *n*-th time level, $I_{n}=\left[t_{n},t_{n+1}\right)$ be the update time interval slab, and $\Delta t_{n+1}$ be the time-step size. We now define our space–time finite element spaces on slab $I_{n}$ as follows:

$$V_{h}^{p,l,n}=\left\{v\in {\left(L^{2}\left(\Omega \times I_{n}\right)\right)}^{d}:v{|}_{K\times I_{n}}\in {\left(P_{p}\left(K\right)\right)}^{d}⊗\left(P_{l}\left(I_{n}\right)\right),\forall K\in T_{h}\right\}\;,$$

$$W_{h}^{p,l,n}=\left\{w\in L^{2}\left(\Omega \times I_{n}\right):w{|}_{K\times I_{n}}\in P_{p}\left(K\right)⊗P_{l}\left(I_{n}\right),\forall K\in T_{h}\right\}\;,$$

$$M_{h}^{p,l,n}=\left\{\mu \in L^{2}\left(E_{h}\times I_{n}\right):\mu {|}_{F\times I_{n}}\in P_{p}\left(F\right)⊗P_{l}\left(I_{n}\right),\forall F\in E_{h}\right\}\;,$$

where the time integrator l≥0, $P_{p}\left(K\right)$ is the space of polynomials of degree at most *p* on cell *K*, and *F* are the edges in $E_{h}$. For clarity, we define the following notation for functions in time slab $I_{n}$ as follows:

$$\zeta^{n,+}:=\lim_{t↘t_{n}}\zeta^{n}\left(t,x\right)\;,\;\zeta^{n,-}:=\lim_{t↗t_{n+1}}\zeta^{n}\left(t,x\right)\;.$$

The DG temporal discretization is performed by integrating Equation (9c) over time slab $I_{n}$. We then perform integration by parts twice and choose a test variable $\psi_{i}$ that vanishes outside of $I_{n}$. For the other equations, Equations (9a), (9b) and (9d), we use an atypical Gauss–Radau quadrature rule. We have one equation for points $I_{n}=\left[t_{n},t_{n+1}\right)$ and one equation for point $t_{n+1}$.

We can formally state our scheme, HDG for the spatial discretization and DG for the temporal discretization, as follows: for i=1,2,⋯,N, we seek an approximation $(\omega_{i,h}^{n},L_{h}^{n},u_{i,h}^{n},\phi_{h}^{n},{\hat{u}}_{i,h}^{n},{\hat{\phi }}_{h}^{n})\in$$V_{h}^{p,l,n}\times V_{h}^{p,l,n}\times W_{h}^{p,l,n}\times W_{h}^{p,l,n}\times M_{h}^{p,l,n}\times M_{h}^{p,l,n}$, such that for t>0,

(A1a) $$\begin{array}{c} \sum_{K\in T_{h}}\left(\int_{I_{n}}\int_{K}k_{i}^{-1}\omega_{i,h}^{n}b_{i}\;dxdt\right.\\ \left.-\int_{I^{n}}\int_{K}\left(u_{i,h}^{n}+\frac{z_{i}e}{k_{B}T}\phi_{h}^{n}\right)\nabla \cdot b_{i}\;dxdt\right)=0\;,\\ \forall b_{i}\in V_{h}^{p,l,n}\;, \end{array}$$

(A1b) $$\begin{array}{c} \sum_{K\in T_{h}}\left(\int_{K}k_{i}^{-1}\omega_{i,h}^{n,-}b_{i}^{n,-}\;dx\right.\\ \left.-\int_{K}\left(u_{i,h}^{n}+\frac{z_{i}e}{k_{B}T}\phi_{h}^{n}\right)\nabla \cdot b_{i}^{n,-}\;dx\right)=0\;,\\ \forall b_{i}^{n,-}\in V_{h}^{p}\;, \end{array}$$

(A1c) $$\begin{array}{c} \sum_{K\in T_{h}}\left(\int_{I_{n}}\int_{K}L_{h}^{n}G\;dxdt-\int_{I^{n}}\int_{K}\phi_{h}^{n}\nabla \cdot G\;dxdt\right.\\ \left.+\int_{I^{n}}\int_{\partial K}{\hat{\phi }}_{h}^{n}G\cdot n\;dsdt\right)=0\;,\;\forall G\in V_{h}^{p,l-1,n}\;, \end{array}$$

(A1d) $$\begin{array}{c} \sum_{K\in T_{h}}\left(\int_{K}L_{h}^{n,-}G^{n,-}dx-\int_{K}\phi_{h}^{n,-}\nabla \cdot G^{n,-}dx\right.\\ \left.+\int_{\partial K}{\hat{\phi }}_{h}^{n,-}G^{n,-}\cdot n\;ds\right)=0,\;\forall G\in V_{h}^{p}, \end{array}$$

(A1e) $$\begin{array}{c} \sum_{K\in T_{h}}\left(\int_{I_{n}}\int_{K}\frac{\partial \exp(u_{i,h}^{n})}{\partial t}\psi_{i}dxdt+\int_{K}\left(\exp\left(u_{i,h}^{n,+}\right)-\exp\left(u_{i,h}^{n-1,-}\right)\right)\psi_{i}^{n,+}dx\right.\\ \left.-\int_{I_{n}}\int_{K}\omega_{i,h}^{n}\nabla \psi_{i}dxdt+\int_{I_{n}}\int_{\partial K}\omega_{i,h}^{n}\cdot n\left(\psi_{i}-{\hat{\psi }}_{i}\right)dsdt\right.\\ \left.+\int_{I_{n}}\int_{\partial K}\left(\eta \alpha_{i}\left(u_{i,h}^{n}-{\hat{u}}_{i,h}^{n}\right)+\eta \xi_{i}\left(\phi_{h}^{n}-{\hat{\phi }}_{h}^{n}\right)\right)\left(\psi_{i}-{\hat{\psi }}_{i}\right)\;dsdt\right)=0\;,\;\\ \forall \psi_{i}\in W_{h}^{p,l,n}\;, \end{array}$$

(A1f) $$\begin{array}{c} \sum_{K\in T_{h}}\left(-\int_{I_{n}}\int_{K}\varepsilon L_{h}^{n}\nabla vdxdt+\int_{I_{n}}\int_{\partial K}\left(\varepsilon L_{h}^{n}\cdot n+\eta \varepsilon \left(\phi_{h}^{n}-{\hat{\phi }}_{h}^{n}\right)\left(v-\hat{v}\right)\right)dsdt\right.\\ \left.-\int_{I_{n}}\int_{K}\left(\rho_{0}+\sum_{i=1}^{N}z_{i}e\exp\left(u_{i,h}^{n}\right)\right)v\;dxdt\right)=0\;,\;\forall v\in W_{h}^{p,l-1,n}\;, \end{array}$$

(A1g) $$\begin{array}{c} \sum_{K\in T_{h}}\left(-\int_{K}\varepsilon L_{h}^{n,-}\nabla v^{n,-}dx\right.\\ \left.+\int_{\partial K}\left(\varepsilon L_{h}^{n,-}\cdot n+\eta \varepsilon \left(\phi_{h}^{n,-}-{\hat{\phi }}_{h}^{n,-}\right)\left(v^{n,-}-{\hat{v}}^{n,-}\right)\right)ds\right.\\ \left.-\int_{K}\left(\rho_{0}+\sum_{i=1}^{N}z_{i}e\exp\left(u_{i,h}^{n,-}\right)\right)v^{n,-}\;dx\right)=0\;,\;\forall v\in W_{h}^{p}\;. \end{array}$$

We note that the software NGSolve that we use for implementation currently does not support HDG in space and DG in time discretization. We leave the numerical simulation implementation of Equation (Appendix A) and the analysis on convergence for future work.
